# Diversity of viral photosystem-I *psaA* genes

**DOI:** 10.1038/ismej.2014.244

**Published:** 2014-12-23

**Authors:** Gur Hevroni, Hagay Enav, Forest Rohwer, Oded Béjà

**Affiliations:** 1 Faculty of Biology, Technion—Israel Institute of Technology, Haifa, Israel; 2 Department of Biology, San Diego State University, San Diego, CA, USA

## Abstract

Marine photosynthesis is one of the major contributors to the global carbon cycle and the world's oxygen supply. This process is largely driven by cyanobacteria, namely *Synechococcus* and *Prochlorococcus*. Genes encoding photosystem-II (PSII) reaction center proteins are found in many cyanophage genomes, and are expressed during the infection of their hosts. On the basis of metagenomics, cyanophage photosystem-I (PSI) gene cassettes were recently discovered with two gene arrangements *psaJF→C→A→B→K→E→D* and *psaD→C→A→B*. It was suggested that the horizontal transfer of PSII and PSI genes is increasing phage fitness. To better understand their diversity, we designed degenerate primers to cover a wide diversity of organisms, and using PCR we targeted the *psaC→A* arrangement, which is unique to cyanophages cassettes. We examined viral concentrates from four islands in the Pacific Ocean and found samples containing the psaC→A arrangement. Analyses of the amplified viral *psaA* gene revealed six subgroups varying in their level of similarity and %G+C content, suggesting that the diversity of cyanophage PSI genes is greater than originally thought.

## Introduction

Marine cyanobacteria of the *Synechococcus* and *Prochlorococcus* genera are significant contributors to global photosynthesis (Li *et al.*, 1993; Liu *et al.*, 1997; Partensky *et al.*, 1999). It was shown that a number of viruses (cyanophages) that infect these cyanobacteria carry photosynthetic genes coding for photosystem-II (PSII) proteins (Mann *et al.*, 2003; Lindell *et al.*, 2004; Millard *et al.*, 2004; Lindell *et al.*, 2005; Sullivan *et al.*, 2005; Sullivan *et al.*, 2006; Sharon *et al.*, 2007) and photosystem-I (PSI) proteins (Sharon *et al.*, 2009; Alperovitch-Lavy *et al.*, 2011; Béjà *et al.*, 2012).

**Figure 1:**
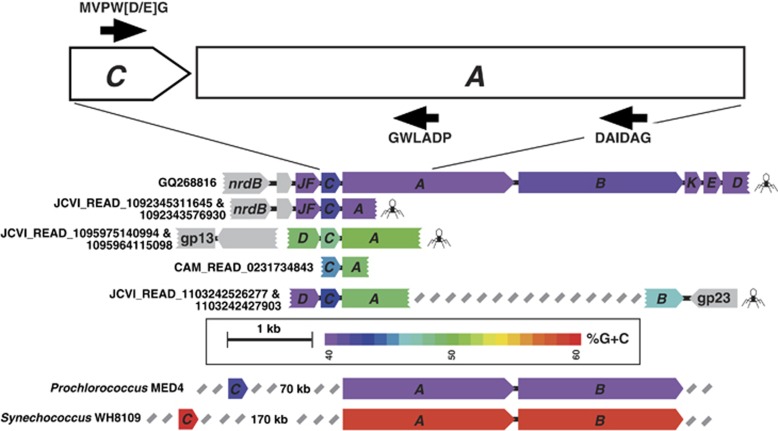
Schematic physical maps of viral long PCR, viral GOS sequences and cyanobacteria containing PSI gene cassettes. PSI genes are colored according to their %G+C content. Gray arrows represent viral open reading frames. Color code indexes indicate %G+C; the calculations were performed for each gene separately. Positions of forward and reverse primers are indicated by black arrows. A phage symbol is attached to each sequence identified as also containing structural viral genes. For space considerations, cyanobacterial gene arrangements are shown only for *Prochlorococcus* Med4 and *Synechococcus* WH8109. The arrangement *psaC* gene followed immediately by a *psaA* gene was not detected in any of the currently available cyanobacterial genomes (37 species in CyanoBase (http://genome.microbedb.jp/cyanobase); November 2014).

Three viral PSI gene organizations are currently known: a single *psaJ* gene (Sharon *et al.*, 2011), and two gene cassettes *psaJF→C→A→B→K→E→D* and *psaD→C→A→B* (Sharon *et al.*, 2009; Alperovitch-Lavy *et al.*, 2011; Béjà *et al.*, 2012). Although the longer cassette is readily detected in more than 20 Global Ocean Sampling expedition (GOS) (Rusch *et al.*, 2007)) scaffolds (Sharon *et al.*, 2009; Alperovitch-Lavy *et al.*, 2011; Béjà *et al.*, 2012)], fragments from the short cassette were detected only three times in different metagenomic data sets (Béjà *et al.*, 2012). In addition to differences at the protein level, *psaA* genes coming from the *psaD→C→A→B* gene organization are distinguishable in their higher %G+C content compared with *psaA* genes coming from the long viral gene organization (∼50%G+C and ∼40%G+C, respectively; Figure 1).

Cyanophages containing the *psaJF→C→A→B→K→E→D* gene organization were hypothesized to produce a monomeric PSI (as opposed to a trimeric PSI observed in their cyanobacterial hosts and similarly to what is observed in plants) and to shift host metabolism toward a cyclic photosynthetic mode (Sharon *et al.*, 2009; Philosof *et al.*, 2011). Therefore, infected cells are assumed to keep on harvesting light energy while avoiding the fixation of CO_2_. Hence, the gained energy (in the form of ATP) is used to enhance nucleotide biosynthesis, required for the replication of additional viral genomes (Sharon *et al.*, 2009; Philosof *et al.*, 2011; Thompson *et al.*, 2011; Enav *et al.*, 2014). Current data about the *psaD→C→A→B* arrangement is so scarce that no working hypothesis have been put forward yet beside the mentioning that an ancestral PSI composed of only these four subunits is a plausible scenario (Nelson, 2011).

The gene combination *psaC→psaA* is present in both the long and short viral cassettes, however, it is absent in their potential cyanobacterial marine hosts *Prochlorococcus* and *Synechococcus* (Figure 1). Therefore, in order to better understand the diversity of viral PSI genes, we used general degenerate primers designed against PsaC and PsaA proteins to amplify specifically viral *psaC→psaA* amplicons from different environmental samples.

## Materials and methods

### *PsaC*-*PsaA* gene amplification and cloning

Degenerate primers were designed against the PSI PsaC and PsaA proteins (Primers PsaCdeg-fwd, PsaAdeg2-rev and PsaA-rev with 64 128 and 256 degeneracy, respectively; see Supplementary Table S1 for primer sequences) based on the multiple sequence alignment of cyanobacterial and viral proteins obtained from the GOS data set (Rusch *et al.*, 2007) and viral PSI proteins previously discovered (Sharon *et al.*, 2009; Béjà *et al.*, 2012). PCR reactions using primers to target the *psaC→A* gene arrangement (Figure 1; each reaction contained a forward primer and one of the reverse primers) were performed directly on viral concentrates from the Pacific Southern Line Islands (collected in April 2009 from the Millennium, Flint, Malden and Starbuck Islands) (Supplementary Figure S1; see Supplementary Tables S1 and 2 in Kelly *et al.* (2014) for metadata details on samples Millennium 9, Flint 6, Malden5 and Starbuck 7). Viral concentrates were prepared according to Dinsdale *et al.* (2008). Each PCR reaction was performed using BIO-X-ACTShort mix (Bioline, London, UK). PCR amplification was carried out in a total volume of 30* *μl containing 1* *μl of phage concentrate as template, 0.8 mM dNTPs, 2 mM MgCl2, 1 μM primers (each) and 2.4 U BIO-X-ACTShort DNA polymerase. The amplification conditions included the steps at 95 °C for 5 min, 40 cycles of 95 °C for 30 s, 56 °C for 30 s and 72 °C for 1 min. PCR products (450–530 bp with the inner reverse primer and 850 bp with the outer reverse primer) were subcloned using the QIAGEN (Hilden, Germany) PCR cloning kit according to the manufacturer's specifications and sequenced using Sanger sequencing (Macrogen Europe, Amsterdam, NL, USA).

### Tetranucleotide frequency analysis

In order to profile the genomic composition of different *psaA* genes, each *psaA* sequence was represented by a tetranucleotide frequency vector (256 features per vector). Frequencies were calculated exclusively from the coding strand using overlapping windows (window location was modified by single-nucleotide steps) and normalized to the sequence length (See Supplementary file S1 for the alignment).

To discover possible differences between the genomic compositions of *psaA* genes, principal component analysis was performed using tetranucleotide frequency vectors as the input value matrix. The three most variable principal components were plotted using GNU R RGL and ColorRamp packages (Keitt, 2008).

To better define the similarities and differences between the groups of *psaA* genes we calculated the cosine distance for each of the two genomic profiles (equation 1). We generated a symmetrical distance matrix of cosine similarities between all the genomic profiles and clustered the matrix using MultiExperiment Viewer (MeV) software (Saeed *et al.*, 2006; hierarchical clustering method set to Pearson's correlation, average linkage).


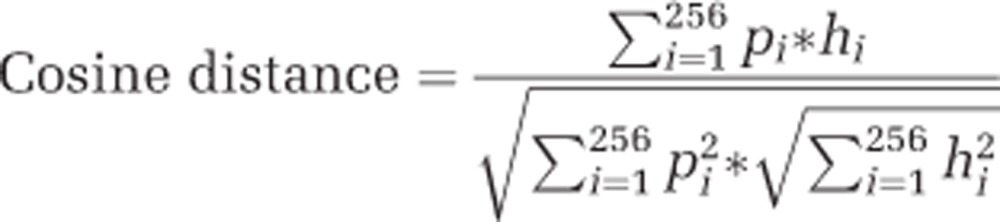


### PsaA phylogenetic tree construction and analysis

PCR products obtained were trimmed to include only the *psaA* gene portion (PsaC proteins are too conserved to be used as phylogenetic markers) and translated according to the correct open reading frame. PsaA protein sequences from the PCR products and reference sequences from the GOS project (downloaded from the CAMERA website (http://camera.crbs.ucsd.edu/projects/)), and various *Prochlorococcus* and *Synechococcus* (downloaded from GenBank) were then aligned using Muscle under default parameters (Edgar, 2004). Maximum-likelihood phylogenetic trees were constructed using the phylogeny.fr pipeline (Dereeper *et al.*, 2008), with PhyML v 3.0 (Guindon *et al.*, 2010) and the WAG substitution model for amino acids (Whelan and Goldman, 2001). Five hundred bootstrap replicates were conducted for each analysis. Trees were visualized using FigTree v 1.4.0 (http://tree.bio.ed.ac.uk/software/figtree/). See Supplementary file S2 for the alignment used to construct the tree.

### Analyses of Southern Line Islands microbiomes

The microbial metagenomes of the samples used in this study for PCR have been previously deposited in the MG-RAST Metagenomics Analysis Server (Meyer *et al.*, 2008), http://metagenomics.anl.gov/linkin.cgi?project=9220 (project name: Pacific Reef Microbiomes; Kelly *et al.*, 2014). MG-RAST automated platform analyses were used in order to calculate the proportions of different cyanobacterial groups in these stations.

### Shannon entropy

Shannon entropy was calculated using the Shannon Entropy-One tool from the HIV database (http://www.hiv.lanl.gov/content/sequence/ENTROPY/entropy_one.html). Reported values are summation of the position-specific values outputted by the tool.

## Results and Discussion

**Figure 2:**
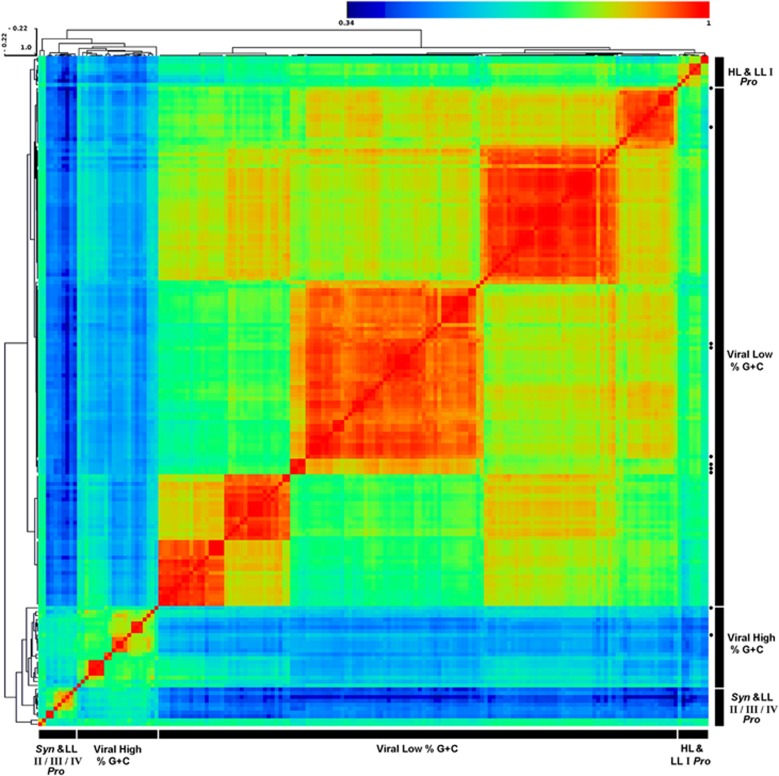
Distance matrix based on tetranucleotide frequencies of *psaA* gene sequences from GOS, *Prochlorococcus*, marine *Synechococcus* and PCR amplicones from the viral concentrates. Colors range from red, indicating high similarity, to blue, indicating higher distance. Black dots represent reported sequences from the GOS expedition. *Synechococcus* (*Syn*), Low Light adapted (LL) and High Light adapted (HL) *Prochlorococcus* (*Pro*) groups are labelled accordingly. Separation into different LL *Prochlorococcus* groups is according to Scanlan *et al.* (2009). Names were removed for clarity; see Supplementary Figure S2 for full names.

The PsaC forward primer designed based on a conserved domain at the C-terminus of the protein and two reverse primers designed against the PsaA protein were used in order to perform PCR reactions that will amplify viral-only gene arrangements (Figure 1 and Supplementary Table S1). Using these primers, PCR was performed directly on viral concentrates collected from the remote Pacific Line Islands (see Map in Supplementary Figure S1), where high proportions of cyanophages containing PSI genes were reported (Sharon *et al.*, 2009). Tetranucleotide frequency analysis of the amplified environmental viral *psaA* genes (that is, the *psaA* gene from the *psaCA* amplicon) revealed six viral *psaA* subgroups (Figure 2, and PCA movie S1). The optimal number of subgroups was determined using pseudo-F statistic with 100 iterations (Caliński and Harabasz, 1974). One group was composed of ∼50%G+C *psaA* amplicons whereas the other five groups contained ∼40%G+C *psaA* amplicons. Only 17% of the amplified *psaA* sequences were closely similar to the few previously reported GOS viral *psaA* sequences (⩾97% on the nucleotide level). Shannon entropy measures (Supplementary Table S2) show that the diversity of the viral high- and low-%G+C *psaA* groups is higher than what is observed in the *Synechococcus* and HL *Prochlorococcus* groups. Although the LL *Prochlorococcus* share the same ecological niche, the observed high Shannon entropy is a result of different %G+C observed within LL *Prochlorococcus* groups.

Overall, 12% of the clones were from the 50%G+C *psaA* cluster, however, the proportion markedly changed between the stations (Malden 25% (*n*=20), Millennium 19% (*n*=61), Starbuck 1.9% (*n*=51) and Flint 0% (*n*=7)). We speculate that this indicates on the availability of certain cyanobacterial hosts, which were present at the time of sampling in the Malden and Millennium Islands. Malden and Millennium islands had higher proportions of marine *Synechococcus* versus *Prochlorococcus* in the metagenomic data (73% *Synechococcus*, on the genus level), which might be explained by the higher proportions of nitrite and nitrate compared with ammonium observed in these stations (Moore *et al.*, 2002; (see Supplementary Table S1 in Kelly *et al.*( 2014)). In contrast, different cyanobacterial proportions are observed in Starbuck and Flint islands (34% and 54% *Synechococcus,* respectively). However, we could not correlate any specific *Synechococcus* strains with the high-%G+C *psaCA* gene organization.

**Figure 3:**
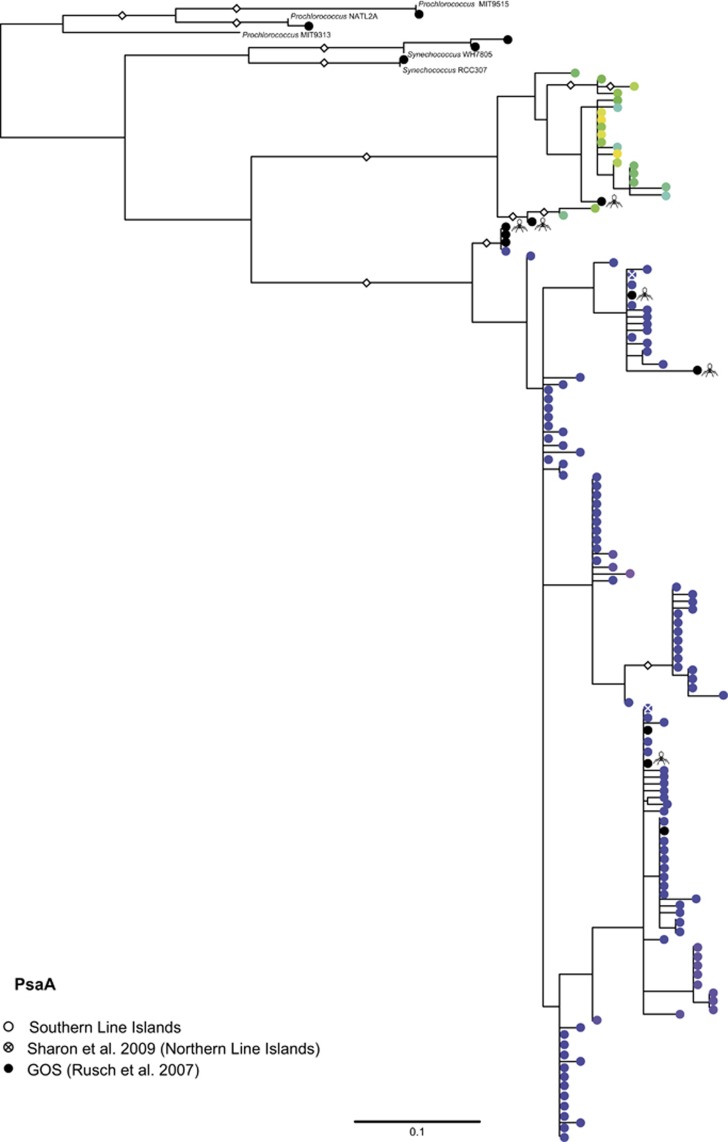
PsaA phylogenetic tree. Diamond symbols represent bootstrap values higher than 70%. Circle color represents %G+C classification according to the color index in Figure 1: green and yellow—High (∼50%), purple—Low (∼40%). Black dots represent sequences from the GOS expedition. A phage symbol is attached to each GOS sequence identified as also containing structural viral genes. The scale bar indicates the average number of amino-acid substitutions per site. Names were removed for clarity; see Supplementary Figure S3 for full names.

At the protein level, the environmental PsaA proteins were separated into two statistically supported clusters (Figure 3), with each cluster composed of PsaA proteins predicted to originate either from the *psaJF→C→A→B→K→E→D* or *psaD→C→A→B* gene organizations (predicted based on %G+C content of the corresponding genes). The separation into two distinct protein groups could either be the result of simply being operated in different hosts (that is, *Prochlorococcus* or marine *Synechococcus*), or could indicate operation at different photosynthetic modes in similar hosts (for example, working in a cyclic mode or in monomeric or trimeric PSI complexes (Sharon *et al.*, 2009; Alperovitch-Lavy *et al.*, 2011; Philosof *et al.*, 2011)).

The use of PCR primers designed to amplify a specific gene arrangement enabled the screening for this unique viral *psaCA* gene combination without the need to clean the sample from other possible gene arrangements that might react with the primers, but are separated by a long DNA stretch or are in the wrong orientation (such as those found in various *Prochlorococcus* or marine *Synechococcus*). We have not ruled out the possibility that the unique *psaCA* gene organizations we obtained by PCR, originate from yet uncultured cyanobacteria. However, based on the existence of viral genes on the GOS scaffolds with similar gene organization and the amplification from viral concentrates, we find this scenario highly unlikely.

Our simple yet efficient targeted screen of viral photosynthetic genes revealed greater diversity of these genes than anticipated. Following the discovery of the single *psaJ* gene (Sharon *et al.*, 2011) and the two currently known PSI gene cassettes (Sharon *et al.*, 2009; Alperovitch-Lavy *et al.*, 2011; Béjà *et al.*, 2012), this new finding may suggest that other viral PSI gene arrangements and modes of photosynthetic operation are awaiting discovery.
