# Supplementary material for: Diversity of viral photosystem-I psaA genes
Source: ISME J. 2014 Dec 23;9(8):1892–8. doi: 10.1038/ismej.2014.244 (PMC4511924; doi:10.1038/ismej.2014.244)
Supplement: Supplementary Table 1 [file ismej2014244x1.doc]

**Supplementary Table S1. Degenerate primers designed to amplify *psaC-psaA* amplicons**

| **Name** | **Primers** | **Corresponding peptide** | **Reference** |
| --- | --- | --- | --- |
| PsaCdeg-fwd | (5’-ATGGTNCCNTGGGANGG-3’) | MVPW[D/E]G | this study |
| PsaAdeg2-rev | (5’-CCNGCRTCDATNGCRTC-3’) | DAIDAG | this study |
| PsaA-rev | (5’-GGRTCNGCNARCCANCC-3’) | GWLADP |  |
