# Supplementary material for: Diversity of viral photosystem-I psaA genes
Source: ISME J. 2014 Dec 23;9(8):1892–8. doi: 10.1038/ismej.2014.244 (PMC4511924; doi:10.1038/ismej.2014.244)
Supplement: Supplementary Table 2 [file ismej2014244x2.doc]

| **Group** | **Number of sequences** | **Shannon Entropy** |
| --- | --- | --- |
| Viral Low %G+C | 134 | 65.344 |
| Viral High %G+C | 21 | 75.146 |
| *Synechococcus* | 6 | 55.247 |
| HL *Prochlorococcus* | 6 | 35.63 |
| LL *Prochlorococcus* | 6 | 84.407 |
