# Supplementary material for: Diversity of viral photosystem-I psaA genes
Source: ISME J. 2014 Dec 23;9(8):1892–8. doi: 10.1038/ismej.2014.244 (PMC4511924; doi:10.1038/ismej.2014.244)
Supplement: Supplementary Figure S1 [file ismej2014244x3.pdf]

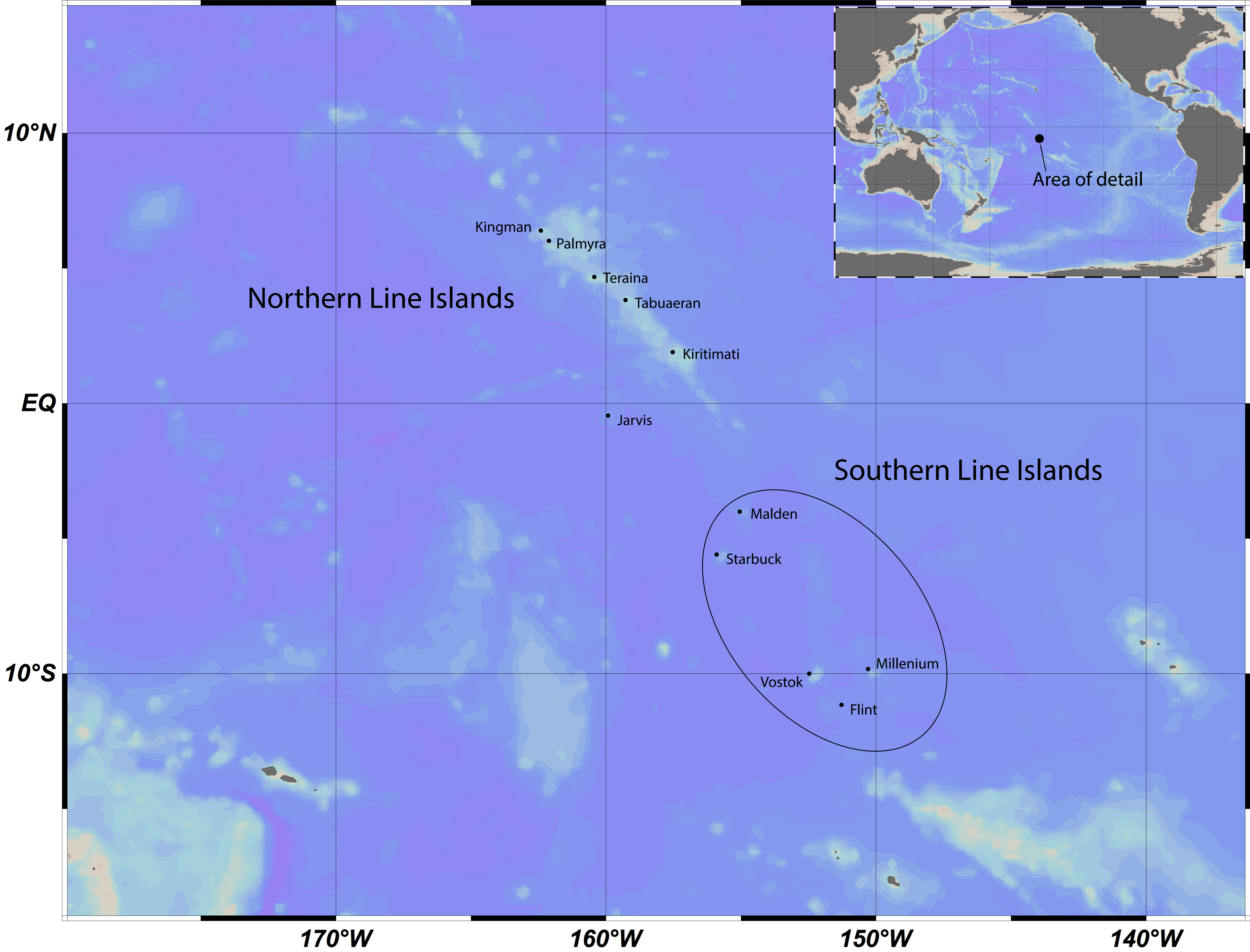

10°N
EQ
10°S
170°W
160°W
150°W
140°W
Area of detail
Kingman
Palmyra
Teraina
Tabuaeran
Kiritimati
Jarvis
Northern Line Islands
Southern Line Islands
Malden
Starbuck
Millenium
Vostok
Flint
