# Supplementary figures and images for: Diversity of viral photosystem-I psaA genes
Source: ISME J. 2014 Dec 23;9(8):1892–8. doi: 10.1038/ismej.2014.244 (PMC4511924; doi:10.1038/ismej.2014.244)

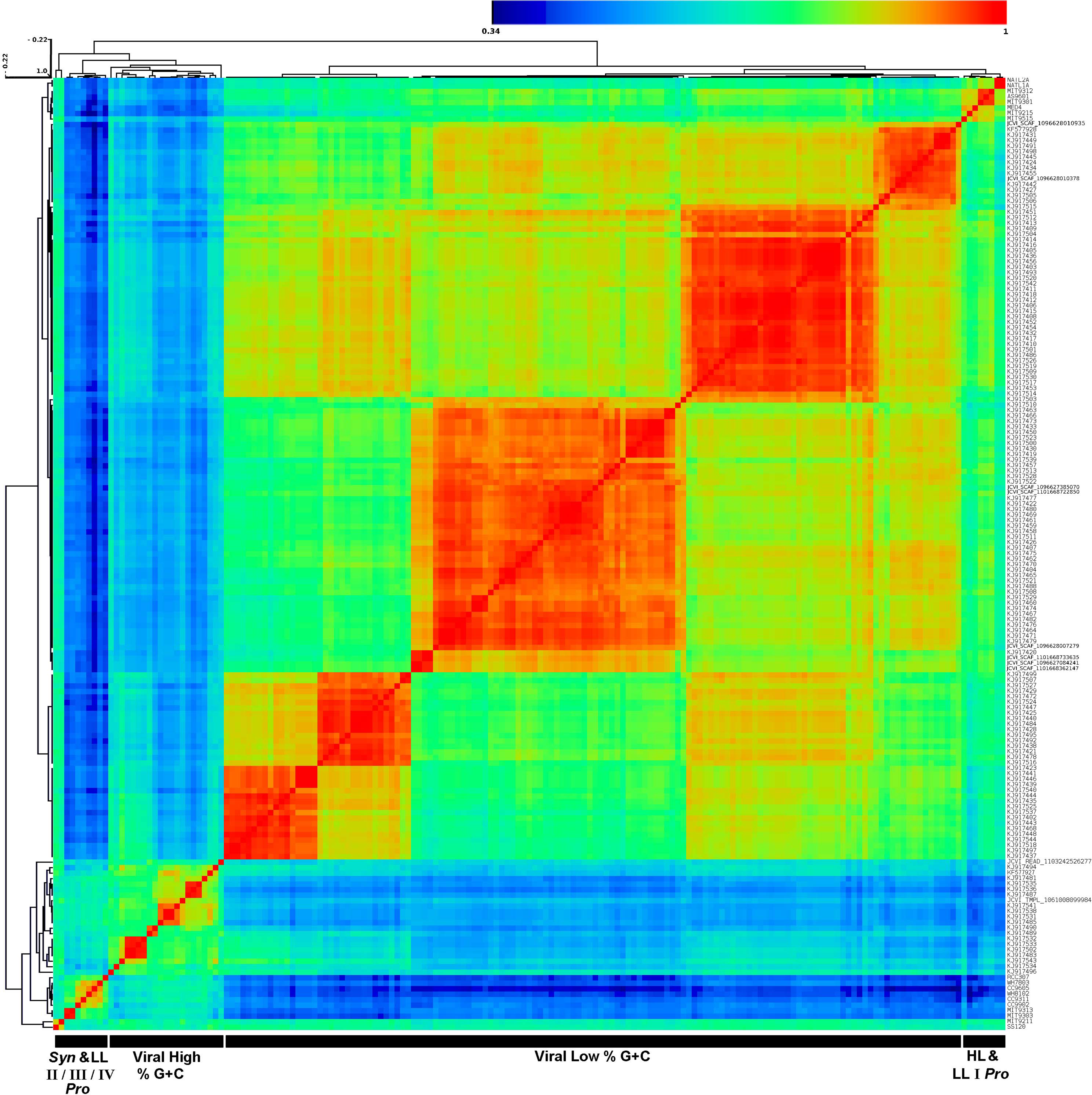

Supplement: Supplementary Figure S2 [file ismej2014244x4.tif]

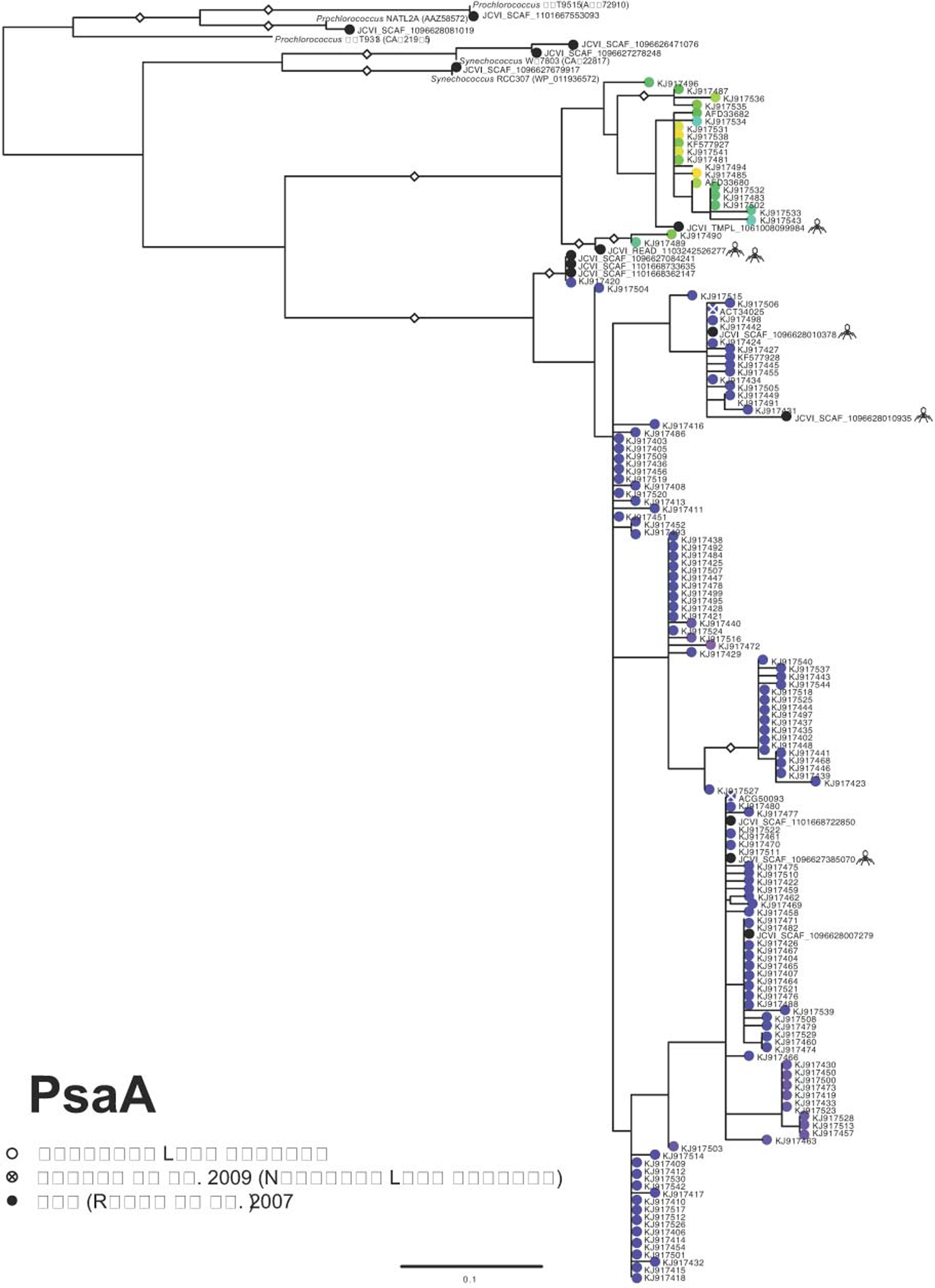

Supplement: Supplementary Figure S3 [file ismej2014244x5.tif]

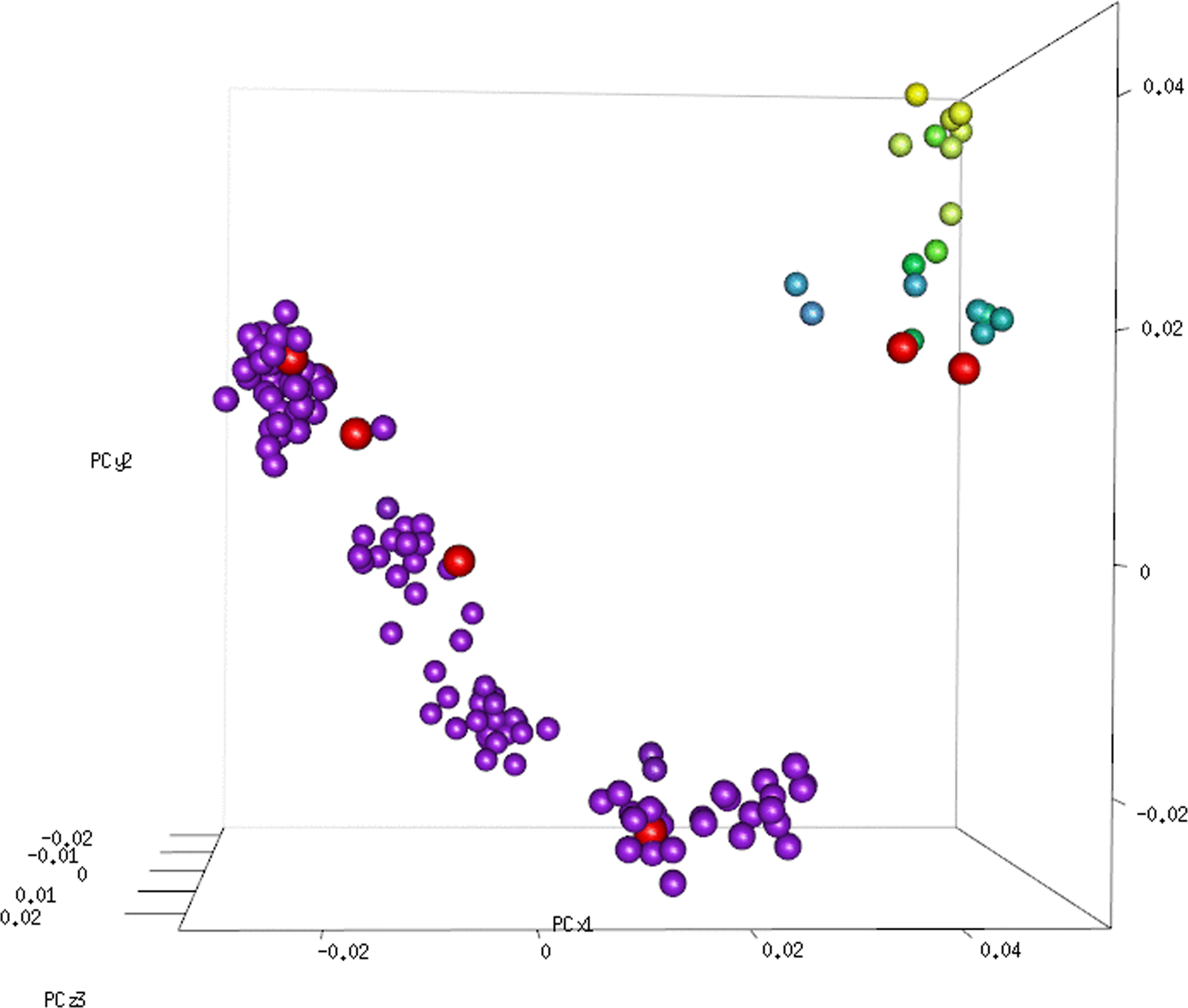

Supplement: Supplementary Figure S4 [file ismej2014244x6.tif]
